# Rectal cancer diagnosed after resection of isolated brain metastasis

**DOI:** 10.1186/s40792-022-01407-8

**Published:** 2022-03-28

**Authors:** Yoshiyuki Tagayasu, Yuji Miyamoto, Hiroshi Sawayama, Katsuhiro Ogawa, Rikako Kato, Naoya Yoshida, Akitake Mukasa, Hideo Baba

**Affiliations:** 1 Department of Gastroenterological Surgery, Graduate School of Medical Sciences, Kumamoto University, 1-1-1 Honjo, Chuo-ku, Kumamoto, 860-8556 Japan; 2 Department of Neurosurgery, Graduate School of Medical Sciences, Kumamoto University, 1-1-1 Honjo, Chuo-ku, Kumamoto, 860-8556 Japan

**Keywords:** Colorectal cancer, Brain metastasis, Surgical resection

## Abstract

**Background:**

Brain metastasis of colorectal cancer is infrequent, and isolated brain metastases are more infrequent. Thus, when neurological symptoms, such as paralysis or disturbance of consciousness appear, there is a high probability that the cancer has spread to other organs.

**Case presentation:**

Here, we present a 64-year-old man with a progressive headache, decreased motivation, and aphasia who was diagnosed with a brain tumor in the left frontal region. He underwent a craniotomy, and the brain tumor was diagnosed as adenocarcinoma. We performed a colonoscopy and diagnosed rectal cancer without other distant metastases. After whole-brain radiotherapy (WBRT), low anterior resection for primary rectal tumor was performed using a robotic system. The patient was discharged in good condition and received postoperative adjuvant therapy for rectal cancer. He showed no signs of recurrence after 1 year of follow-up.

**Conclusions:**

We described a rare case of rectal cancer that was diagnosed after resection of isolated brain metastasis. A good prognosis was achieved with surgery and WBRT.

## Background

The incidence of brain metastasis associated with metastatic colorectal cancer (CRC), although low, is increasing because of more prolonged survival achieved using new systemic chemotherapy [1, 2]. Brain metastases are usually found in late-stage advanced disease, and the vast majority of patients have metastases at other sites. Isolated brain metastases are even more infrequent [3]. Hepatic and lung metastases are common at distant sites. When neurological symptoms, such as paralysis or disturbance of consciousness appear, there is a high probability that the cancer has spread to other organs. Prognosis is, therefore, poor, and palliative treatment is often indicated. Here, we present a case of rectal cancer with isolated brain metastasis, which first developed as a clinical manifestation of insufficiency paralysis and dysarthria.

## Case report

**Fig. 1:**
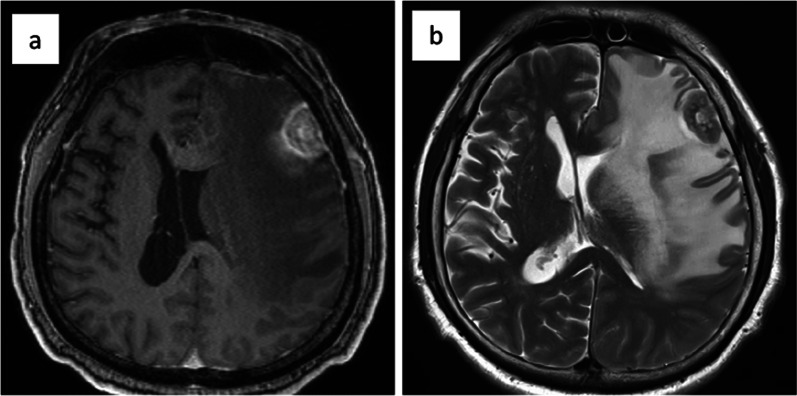
Magnetic resonance imaging of the brain tumor. **a** T1-weighted image showed a nodular lesion (diameter 13 × 26 mm) near the surface of the left frontal lobe. **b** T2-weighted image of a high signal area in the surrounding white matter with median deviation

**Fig. 2:**
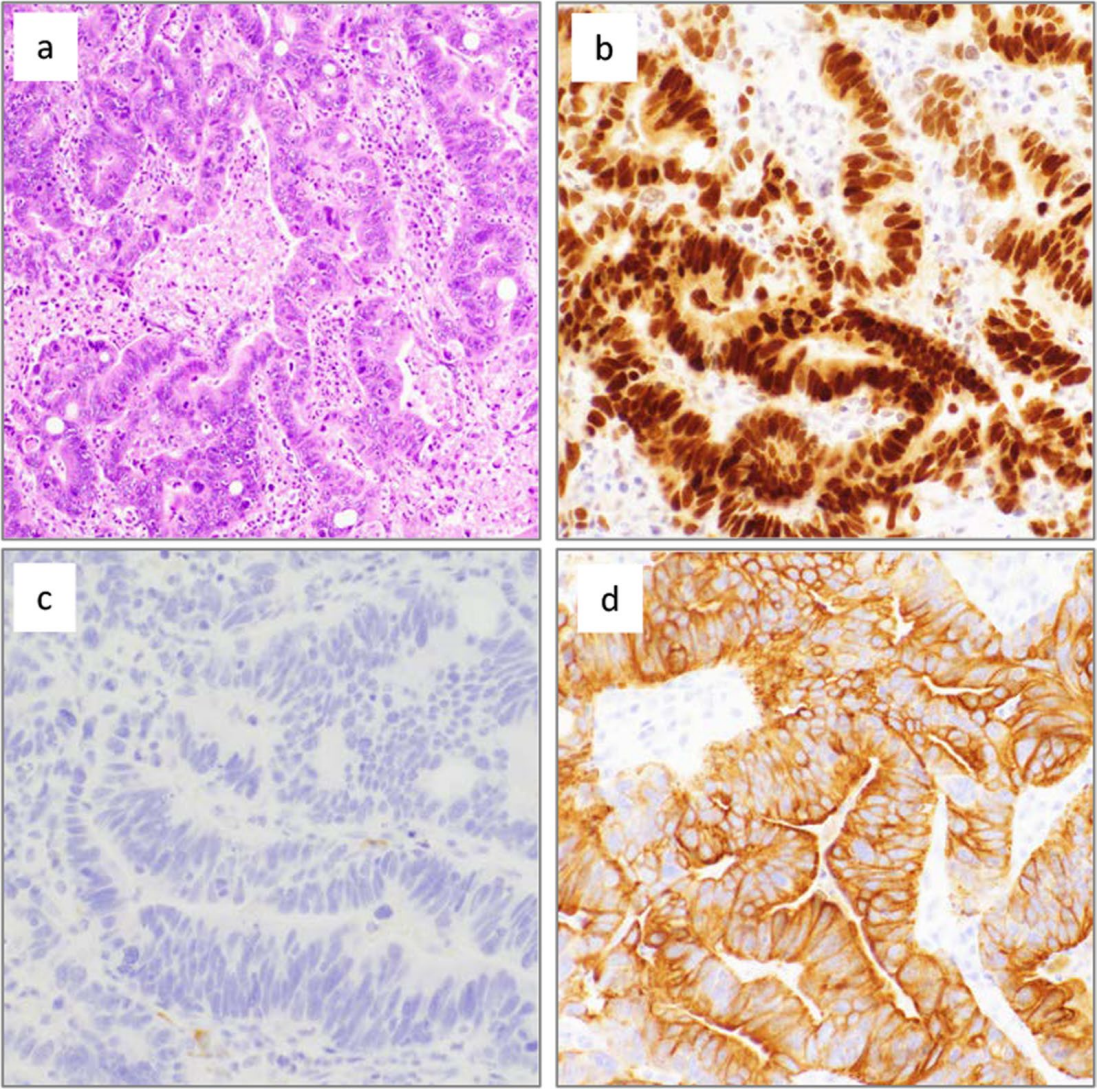
**a** Hematoxylin and eosin staining (200×), **b** Caudal type homeobox 2 (CDX2) (400×), **c** Cytokeratin (CK) 7 (200x), **d** CK20 (400×). These results suggest metastasis from rectal cancer, because immunohistochemical analysis detected CK20 and CDX2 but not CK7

A 64-year-old man with no previous illness visited a local physician because of progressive headache, decreased motivation, and aphasia over 2 weeks. Computed tomography (CT) of the head showed a 3-cm mass in the left frontal region with well-defined borders, irregular margins, and extensive surrounding edema. Contrast-enhanced magnetic resonance imaging of the head strongly suggested a primary brain tumor (Fig. 1). The patient developed right-sided insufficiency paralysis, which we decided to immediately treat by craniotomy. Pathological diagnosis of the brain tumor showed adenocarcinoma. Immunohistochemical analysis detected CK20 and CDX2 but not CK7, suggesting the primary tumor was in the colon (Fig. 2).

**Fig. 3:**
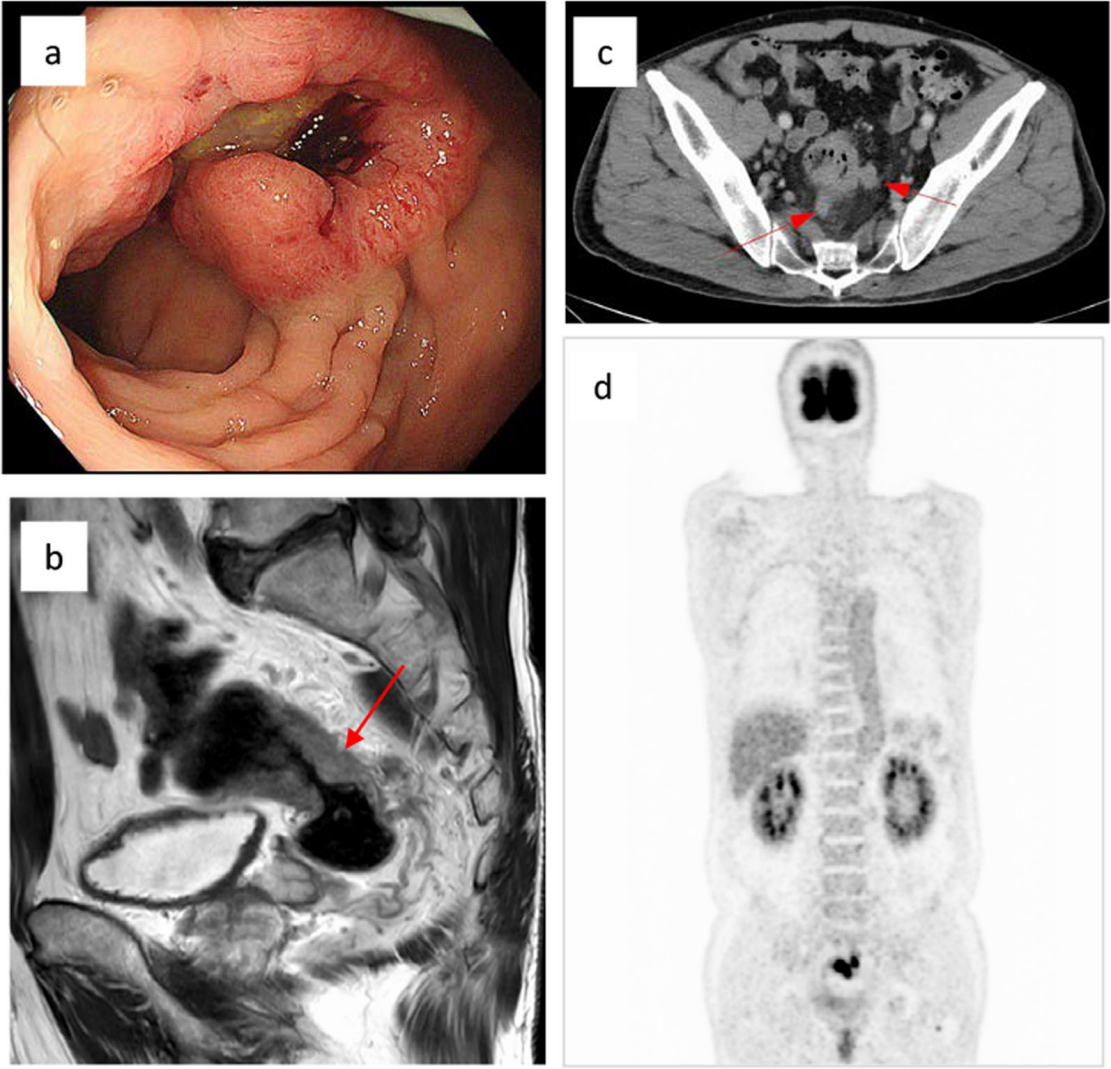
Preoperative image of rectal cancer. **a** Subcircumferential type 2 advanced carcinoma in the lower rectum. **b** Mid-sagittal plane of magnetic resonance imaging analysis of rectal cancer. **c** Abdominal computed tomography (CT) scan of circumferential wall thickening of the rectum and advanced metastasis to regional lymph nodes. **d** Positron emission tomography–CT showed no other obvious distant metastasis

Thus, we performed a colonoscopy 1 week after brain surgery, which revealed a type 2 advanced carcinoma with a circumference of approximately 80% of that of the lower rectum (Fig. 3a). Blood tests were not obviously abnormal, and the carcinoembryonic antigen level did not significantly change before vs after treatment (7.6 ng/ml vs 7.5 ng/ml, respectively). Abdominal CT showed rectal wall thickness and regional lymph node metastases (Fig. 3b, c). However, there was no other obvious distant metastatic site (Fig. 3d). We, therefore, decided to perform curative resection of the lower rectal cancer, staged as cT3N2M1 (TNM classification, 8th edition). After whole-brain irradiation (35 Gy/5 Fr), Twenty-four days after whole brain irradiation (35 Gy/5 fr), we performed robotic low anterior resection using the da Vinci Surgical System. Pathological findings showed a well-differentiated adenocarcinoma similar to brain tumor tissue (pT3N1aM1 stage IV). The tumor was an invasive growth of moderately differentiated tubular adenocarcinoma that invaded through the muscularis propria into perirectal tissue. The tumor showed vascular invasion (v1b) and lymphatic invasion (Ly1a). The tumor budding had a high score (BD3). Resection margins were negative (R0). High-grade extramural cancer deposits without lymph-node structure (Ex) existed around the primary tumor.

The patient was discharged from the hospital in good condition, and he received capecitabine monotherapy as adjuvant treatment for rectal cancer. The chemotherapy regimen was determined by the patient's preference. Twenty month follow-up after rectal surgery showed no evidence of recurrence.

## Discussion

Brain metastases from CRC are uncommon, occurring in 0.4–5.4% of cases [2, 4]. The increasing incidence of brain metastases in patients with metastatic CRC has been attributed to the longer survival seen with newer systemic therapies [1, 5]. Thus, brain metastases are generally found as a part of systemic diseases. In addition, brain metastasis is generally considered to have a poor prognosis, with survival expectancy < 6 months. For example, Farnell et al. reported that the 1-year survival rate of patients with brain metastases is 16% after diagnosis, and the median survival time is 42 weeks after surgical resection plus postoperative radiotherapy [6–8].

As in our case, solitary brain metastasis detected prior to primary CRC diagnosis is very rare. Several cases have reported discovery of brain metastases before diagnosis of primary colonic cancer [9, 10]; however, only one case of isolated brain metastases has been reported [11].

Previous studies have suggested that there is a relationship between brain anatomy and brain metastasis [12, 13]. Brain metastasis preferentially arises at the cerebellum, gray–white matter junction, and watershed areas. In our case, the tumor was located near the surface of the left frontal lobe, which was one of the reasons why resection was chosen.

The primary approaches to the treatment of brain metastases include surgery, stereotactic radiosurgery, and whole-brain radiotherapy (WBRT). Important factors to consider in patients presenting with a single brain mass suspected of being a metastatic tumor include: tumor size and location; degree of mass effect and edema; presence or absence of symptoms; functional status and extent of systemic disease; and patient preferences with regard to invasive therapy. In the present case, neurosurgical resection was performed first, as a response to rapidly progressing neurological symptoms. The subsequent pathological diagnosis indicated CRC. Surgery is generally indicated for treatment of brain metastases if survival is expected to be at least a few months, if the tumor is resectable, and if other metastatic sites are under control. Furthermore, therapy after resection of brain metastases reduces intracerebral recurrence [14]. Here we performed postoperative WBRT, which controlled recurrence at the time this manuscript was accepted.

In our case, the patient received WBRT after brain metastasis resection, which is considered controversial [15]. Three randomized clinical trials have compared surgery plus WBRT with WBRT alone in patients with single brain metastases. Two of these demonstrated a survival benefit and indicated which patients can benefit from this combined approach, such as young patients and those with solitary brain metastasis, without extracranial metastasis, as in our case.

Recently, stereotactic irradiation of the brain tumor has been developed with local control rates of 80–90% [16]. Japanese guidelines recommend stereotactic irradiation when the number of brain metastases is no more than three or four and the maximum diameter of each metastasis does not exceed 3 cm [17]. In general, patients with brain metastases have a poor prognosis. Although the primary tumor is immediately resected after brain metastasis, new metastases may appear early in the course of the disease. If there are no symptoms related to the primary tumor, then systemic chemotherapy and follow-up may be considered. In our case, there were no symptoms related to the primary tumor, but since no evident metastasis was found in the systemic examination after brain tumor resection, we decided to perform surgery. In addition, the fact that the patient was taking anticoagulants was another reason for performing primary tumor resection immediately.

## Conclusions

We have reported a rare case of rectal cancer that was diagnosed after resection of isolated brain metastasis. Although there was no solid evidence for brain metastasis of CRC, our patient had a good prognosis with surgery and WBRT.

## Data Availability

All data generated or analyzed during this study are included in this published article.

## Abbreviations

CRC: Colorectal cancer
CT: Computed tomography
WBRT: Whole-brain radiotherapy
